# Involvement of DNA ligase III and ribonuclease H1 in mitochondrial DNA replication in cultured human cells

**DOI:** 10.1016/j.bbamcr.2011.08.008

**Published:** 2011-12

**Authors:** Heini Ruhanen, Kathy Ushakov, Takehiro Yasukawa

**Affiliations:** a The Wolfson Institute for Biomedical Research, University College London, Gower Street, London, WC1E 6BT, UK; b Consortium for Mitochondrial Research (CfMR), University College London, UK

**Keywords:** Mitochondrion, Mitochondrial DNA, DNA replication, Mitochondrial DNA replication factor, Okazaki fragment

## Abstract

Recent evidence suggests that coupled leading and lagging strand DNA synthesis operates in mammalian mitochondrial DNA (mtDNA) replication, but the factors involved in lagging strand synthesis are largely uncharacterised. We investigated the effect of knockdown of the candidate proteins in cultured human cells under conditions where mtDNA appears to replicate chiefly *via* coupled leading and lagging strand DNA synthesis to restore the copy number of mtDNA to normal levels after transient mtDNA depletion. DNA ligase III knockdown attenuated the recovery of mtDNA copy number and appeared to cause single strand nicks in replicating mtDNA molecules, suggesting the involvement of DNA ligase III in Okazaki fragment ligation in human mitochondria. Knockdown of ribonuclease (RNase) H1 completely prevented the mtDNA copy number restoration, and replication intermediates with increased single strand nicks were readily observed. On the other hand, knockdown of neither flap endonuclease 1 (FEN1) nor DNA2 affected mtDNA replication. These findings imply that RNase H1 is indispensable for the progression of mtDNA synthesis through removing RNA primers from Okazaki fragments. In the nucleus, Okazaki fragments are ligated by DNA ligase I, and the RNase H2 is involved in Okazaki fragment processing. This study thus proposes that the mitochondrial replication system utilises distinct proteins, DNA ligase III and RNase H1, for Okazaki fragment maturation.

## Highlights

► The data suggest that DNA ligase III is the replicative DNA ligase in human mitochondria. ► RNase H1 is indispensable for Okazaki fragment processing in human mtDNA replication. ► On the other hand, knockdown of neither DNA2 nor FEN1 affected mtDNA replication. ► Mitochondria utilise distinct proteins for Okazaki fragment maturation from nucleus.

## 1 Introduction

Whilst the majority of cellular DNA is enclosed within the nucleus, mitochondria are also known to contain a separate genome, the mitochondrial DNA (mtDNA). In contrast to diploid nuclear DNA, mtDNA is a multi-copy genome, and in human cells typically 10^3^–10^4^ copies of mtDNA molecules are present per cell. Human mtDNA is composed of closed circular DNA molecules 16,569 base pairs in length, and encodes 13 subunits of the oxidative phosphorylation complexes and 2 ribosomal RNAs and 22 transfer RNAs for translation of the subunits within mitochondria. All other mitochondrial proteins, including those involved in the replication of mtDNA, are encoded by nuclear genes.

For three decades mammalian mtDNA has generally been considered to replicate *via* a characteristic DNA replication mechanism, a strand-displacement model which entails continuous synthesis of both strands of DNA, without the synthesis of short lagging strand fragments, known as Okazaki fragments [1,2]. In this model, virtually all replication intermediates will be partially single-stranded. However, a series of recent studies has demonstrated that mammalian mtDNA replicates *via* two replication modes, neither of which is identical to the strand-displacement model [3–7]. One has many of the properties of conventional coupled leading and lagging strand synthesis in which both nascent strands are composed of DNA (strand-coupled DNA synthesis). The other mode, designated as RITOLS replication [6], is a novel mechanism: whilst the leading strand is composed wholly of DNA, ribonucleotides are incorporated throughout the lagging strand, and only after considerable delay are they replaced by DNA. The strand-displacement model of mtDNA replication [2,8] was suggested to be based on the partially single-stranded replication intermediates that are generated as a result of ribonucleotide loss from the replication intermediates of RITOLS replication [4,6,7]. Although the reason for the presence of the two replication modes for mtDNA is unclear, a substantial fraction of the mtDNA replication intermediates are products of the strand-coupled DNA synthesis mode, and this mode appears to be predominant when cells amplify mtDNA to restore the copy number after transient mtDNA depletion [3,5]. These findings indicate a significant role for the strand-coupled DNA synthesis mode in the maintenance of mtDNA.

In addition to the duplex theta replication mechanisms described above (the strand-coupled DNA synthesis mode and RITOLS replication mode), which were proposed mainly from work in rodent and chick liver and cultured human cells, the operation of a non-theta replication mechanism was recently implicated in the replication of human cardiac mtDNA [9].

The replication of nuclear DNA employs a coupled leading and lagging strand synthesis mechanism in which the lagging strand is made up with Okazaki fragments. The maturation of Okazaki fragments, in which they are joined together to form a continuous nascent strand, requires two sequential steps. The first step is removal of primer RNA, a short stretch of RNA which functions to prime DNA synthesis, called ‘Okazaki fragment processing’. The second step is ligation of the processed 5′ end of the Okazaki fragment and the 3′ end of the next Okazaki fragment. Ribonuclease (RNase) H2, flap endonuclease 1 (FEN1) and the endonuclease/helicase DNA2 are considered to function in the first step [10]. RNase H hydrolyses RNA in the DNA/RNA duplex [11] whilst FEN1 and DNA2 cleave protruding single strands (‘flaps’) from double stranded DNA molecules. Short flaps are cleaved by FEN1 whereas longer flaps are cleaved by DNA2 [10,12]. Three models have been proposed for Okazaki fragment processing: the RNase H/FEN1 model, the DNA2/FEN1 model and the FEN1-only model (see Reference [10]). These models have been based upon experiments using mammalian and yeast nuclear DNA replication systems, however certain aspects of the molecular mechanisms of each processing pathway appear to vary considerably between the two systems. For instance, whilst the nuclear single-stranded DNA binding protein, replication factor A (RPA), stimulates the endonuclease activity of DNA2 and plays an important role in the sequential cleavage of a flap by the two nucleases in the DNA2/FEN1 pathway in yeast, human DNA2 is inhibited by RPA [12]. On the other hand many similarities also exist, such as during the second step of Okazaki fragment maturation in which ligation of the two adjacent Okazaki fragments is conducted by DNA ligase I in both mammals and yeast [13].

The existence of the strand-coupled DNA synthesis replication mode in mammalian mitochondria suggests that the same proteins required for Okazaki fragment maturation in the nucleus, or proteins functionally related to them, must be necessary in these organelles. RNase H1 [14], FEN1 [15], DNA2 [16,17] and DNA ligase III [18] were reported to be present in mammalian mitochondria. RNase H1 knockout mice exhibited embryonic lethality accompanied by prior depletion of mtDNA, implying the necessity of RNase H1 in mtDNA maintenance during development [14]. However, whether and how RNase H1 is involved in the process of DNA synthesis in mtDNA replication is unknown. FEN1 and DNA2 were recently shown by *in vitro* assays to be capable of processing model substrates that mimic intermediate structures in long-patch base excision repair, and possibly in mtDNA replication [16]. It is unknown, however, whether these proteins function synergistically in Okazaki fragment processing in mammalian mitochondria *in vivo*. Regarding DNA ligase, unlike yeast and plant mitochondria [19,20], mammalian mitochondria do not possess DNA ligase I but do contain vertebrate-specific DNA ligase III [18,21]. DNA ligase III participates in DNA repair in the nucleus [21] and was shown to be required for the maintenance of mtDNA [22,23]. Still, a demonstration of whether DNA ligase III is responsible for the ligation step in mtDNA replication remains elusive.

In this study we established an assay system using cultured human cells to investigate the involvement of the potential Okazaki fragment maturation proteins in mtDNA replication. The expression of target proteins was silenced acutely in living cells using short interferencing RNA (siRNA) during the course of transient mtDNA depletion induced with 2′,3′-dideoxycytidine (ddC) and during the following recovery of mtDNA after ddC removal, where mtDNA replication appears to rely heavily on strand-coupled DNA synthesis. Under these conditions, the recovery of mtDNA copy number and the integrity of mtDNA replication intermediates were investigated using real-time quantitative PCR (rt-qPCR) and neutral two-dimensional agarose gel electrophoresis (2D-AGE). Our data indicate that RNase H1 plays an indispensable role in Okazaki fragment processing in human mtDNA replication, and suggest that DNA ligase III joins Okazaki fragments in human mitochondria.

## 2 Material and methods

### 2.1 Cell culture, transfection and ddC treatment

**Fig. 1:**
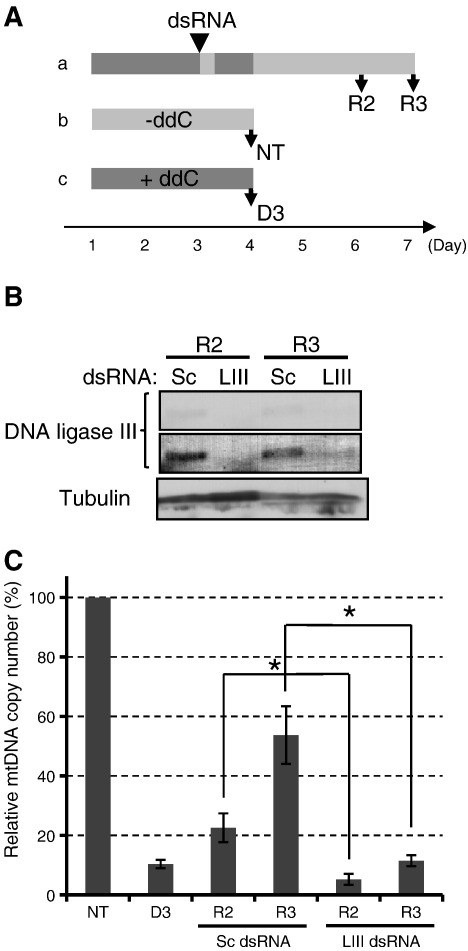
Knockdown of DNA ligase III delays the recovery of mtDNA copy number. (A) Experimental design. The presence and absence of 2′,3′-dideoxycytidine (ddC) in the medium is indicated by dark and pale grey stripes respectively. (a) Time course of the ddC treatment and dsRNA knockdown. R2 and R3 represent the cell harvest points 2 and 3 days after ddC removal, whilst an arrowhead indicates the timing of dsRNA transfection. (b and c) Incubation of cells in medium in the absence or presence of ddC for 3 days as the non-treated or ddC-treated controls. NT and D3 represent the point of their harvest. (B) Western blot analysis of DNA ligase III levels in cell lysates prepared from cells treated with scramble (Sc) dsRNA or DNA ligase III-specific (LIII) dsRNA. The top panel shows the DNA ligase III band and the middle panel is a digitally enhanced image of the top panel. Tubulin was used as a loading control (bottom panel). (C) The relative copy number of mtDNA analysed with real-time quantitative PCR. The mtDNA content was normalised against nuclear gene content in each sample. The relative mtDNA copy number in NT sample was expressed as 100 in each experiment and those of the other samples were displayed relative to this. Data represent the mean of 3 independent transfection experiments ± SEM. * (*p* < 0.05).

The thymidine kinase 1-deficient human osteosarcoma cell line (143B [TK^−^]) was cultured in DMEM (Invitrogen, cat. no. 41966) supplemented with FBS, penicillin–streptomycin and 50 μg/ml uridine (‘normal medium’). The basic procedure of transfection of cells with short double-stranded RNA (dsRNA) occurred as in Ruhanen et al. [24]. Cells were seeded on the day previous to the initial ddC treatment. On day 1 (Fig. 1A), cells were treated with the normal medium containing 25 μM ddC (Sigma) and incubated for 2 days. On day 3, dsRNA transfection was performed with either control scramble (Sc) dsRNA or a gene-specific dsRNA at a concentration of 3 nM using Lipofectamine 2000 reagent (Invitrogen) in Opti-MEM (Invitrogen). In the case of transfection with two separate dsRNA simultaneously, the concentration of each dsRNA was 1.5 nM. Four hours after transfection, DMEM supplemented with 30% FBS, penicillin–streptomycin, 100 μg/ml uridine and 100 μM ddC was added. On day 4, cells were washed with normal medium and further cultured in the normal medium. On days 6 and 7, cells were harvested for analyses. In addition, control cells were simultaneously cultured in the presence or absence of 25 μM ddC for 3 days without dsRNA transfection and harvested. The following dsRNA sequences were used: DNA ligase III; 5′-CUGCAACCCAGAUGAUAUGdTdT-3′ (sense) and 5′-CAUAUCAUCUGGGUUGCAGdTdT-3′ (antisense), RNase H1; 5′-GGAUGGAGAUGGACAUGAA-3′ (sense) and 3′-GACCUACCUCUACCUGUACUU-5′ (antisense), FEN1; 5′-GGAGCGAGCCAAAUGAAGA-3′ (sense) and 3′-CACCUCGCUCGGUUUACUUCU-5′ (antisense), DNA2; 5′-GCAACAACAUGUAUGGGAA-3′ (sense) and 3′-AACGUUGUUGUACAUACCCUU-5′ (antisense). DNA ligase III-specific dsRNA was designed using published information [25] and the synthesis was ordered through Qiagen. Design and synthesis of other dsRNAs were ordered through iGENE Therapeutics, Japan. AllStars Negative Control siRNA (Qiagen) was used as Sc dsRNA. The concentration of the dsRNA solution was based on the information from the manufacturers of dsRNA.

### 2.2 Western blotting

Western blotting was performed as described previously [24] with some modifications. Primary antibodies for DNA ligase III (ab587), RNase H1 (ab56560), FEN1 (ab462), DNA2 (ab96488) and Tubulin (ab56676) were purchased from Abcam (the product codes are indicated in parentheses).

### 2.3 Total DNA preparation and mtDNA quantification

Total DNA was prepared as described previously [24] with modifications. Briefly, cells were lysed with cell lysis buffer (75 mM NaCl, 50 mM EDTA (pH 8), 10 mM Hepes-NaOH (pH 7.2), 1% SDS and 0.2 mg/ml proteinase K) and incubated at 50 °C for 30 min. The sample was extracted sequentially with phenol and chloroform/isoamyl-alcohol, followed by 2-propanol precipitation of DNA. The pellet was rinsed with 70% ethanol, air-dried and dissolved in 10 mM Hepes-NaOH (pH 7.2). Quantification of mtDNA was conducted using a rt-qPCR method essentially as described [24].

### 2.4 DNA modification, 2D-AGE and Southern hybridisation

Total DNA was digested with DraI (New England Biolabs), and then precipitated and suspended in 10 mM Hepes-NaOH (pH 7.2). In some cases the digested DNA was further modified with 9.5 unit S1 nuclease (Promega) in 30 μl reaction mixture at 37 °C for 20 min and then 1.5 μl of 0.5 M EDTA (pH 8) was added to the reaction. The samples were subjected to 2D-AGE essentially as described previously [3], followed by Southern hybridisation as described by Ruhanen et al. [24]. The DNA fragment used for generating the radiolabelled probe covers nt 12,981–13,384 of mtDNA. The probed membranes were exposed to autoradiography film for visualisation of the images and to phosphorimaging plates for quantification of the images with a Typhoon instrument (GE Healthcare).

### 2.5 Statistical analysis

Statistical significance in results was calculated where appropriate using an unpaired Student's *t*-test.

## 3 Results

### 3.1 Knockdown of DNA ligase III delays the recovery of mtDNA copy number after transient depletion of mtDNA

When ddC is added to culture medium within a certain range of low concentrations, ddC specifically inhibits mtDNA replication without apparent toxicity to cells and leads to mtDNA depletion [26]. Upon withdrawal of ddC from the medium, cells amplify mtDNA to restore the copy number. Further, whilst mtDNA replication intermediates in cells continuously cultured in normal medium are a mixture of those produced by strand-coupled DNA synthesis and RITOLS replication, the latter of which requires much less DNA ligation, mtDNA appears to replicate mainly *via* the strand-coupled DNA synthesis mode after the removal of ddC [3,5] (Supplementary Fig. 1). Bearing in mind the considerations outlined above – that is the shift of the mtDNA replication mode towards strand-coupled DNA synthesis and the amplification of mtDNA to restore the copy number after treatment with ddC – we consider that this situation provides an excellent experimental system to investigate the involvement of candidate proteins in the processing and ligation of Okazaki fragments in the strand-coupled DNA replication, and thus knockdown experiments were performed under these conditions using human 143B [TK^−^] cells.

On day 1 culture in medium containing ddC was started, followed by dsRNA transfection on day 3 using LIII dsRNA or control Sc dsRNA. The cells were then further incubated in ddC-containing medium. On day 4, ddC-containing medium was replaced with normal medium, and cells were cultured for another 2 or 3 days before total DNA and total cellular lysates were prepared from them (R2 and R3 respectively, Fig. 1A). Simultaneously, control cells were cultured in the presence or absence of ddC for 3 days, after which total DNA was prepared (Fig. 1A). To preserve mtDNA replication intermediates RNase was not used during DNA isolation, thus the isolates contained RNA.

Western blot analysis of total cellular lysates indicated a strong knockdown of DNA ligase III expression in LIII dsRNA-treated cells (Fig. 1B). Additionally, crude mitochondria were prepared from cells on R2 in separate experiments and subjected to Western blotting. The levels of DNA ligase III in crude mitochondrial preparation were reduced by LIII dsRNA treatment (Supplementary Fig. 2). The copy number of mtDNA was investigated in the total DNA preparation of each sample using rt-qPCR (Fig. 1C). The content of mtDNA in cells exposed to ddC for 3 days (D3) was approximately 10% of that in the non-treated sample (NT), confirming the effective inhibition of mtDNA replication with ddC. Whilst the Sc dsRNA-treated cells showed a similar recovery profile of mtDNA copy number to the cells without dsRNA transfection (Supplementary Fig. 1A), the restoration of mtDNA copy number was delayed in LIII dsRNA-treated cells. This result indicates that DNA ligase III depletion hindered the strand-coupled DNA synthesis under these experimental conditions, presumably due to the delay of ligation of Okazaki fragments.

### 3.2 Sensitivity of mtDNA replication intermediates to S1 nuclease upon DNA ligase III depletion

**Fig. 2:**
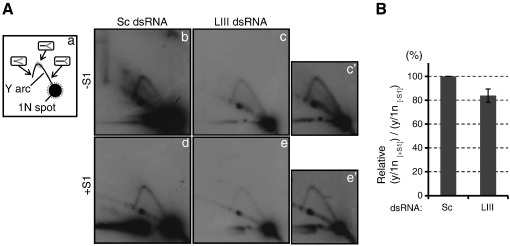
Sensitivity of mtDNA replication intermediates to S1 nuclease upon DNA ligase III depletion. (A) Analysis of a DraI-digested fragment of mtDNA with two-dimensional agarose gel electrophoresis. (a) A schematic drawing of the fragment visualised by Southern hybridisation. The non-replicating molecules (1N spot) and replication intermediates (Y arc) are indicated. The molecule structure of the DraI-digested mtDNA replication intermediates at 3 different positions of Y arc is drawn in the insets with a black bar as non-replicating portion and grey bars as replicated portions of the fragment. The apex region of the Y arc used for the quantification is indicated as a grey rectangle and the region of 1N spot used for the quantification as a grey dotted circle. More information on the fragment is provided in Supplementary Fig. 1B. (b–e) The DraI-digested mtDNA fragment of scramble (Sc) dsRNA or DNA ligase III-specific (LIII) dsRNA-treated cells without (− S1) (b and c) and with S1 nuclease treatment (+ S1) (d and e). The samples in panels b–e were run in the same second dimension gel and panels b–e were produced from an X-ray film. Panels c′ and e′ are a longer exposure version of panels c and e. (B) Numerical presentation of the Y arc stability against S1 nuclease. The value of (y/1n _[+ S1]_)/(y/1n _[− S1]_) from Sc dsRNA-treated samples is expressed as 100 and the relative value from LIII dsRNA-treated samples was calculated. Data represent the mean of 3 independent transfection experiments ± SEM. Sensitivity of mtDNA replication intermediates to S1 nuclease upon DNA ligase III depletion. (A) Analysis of a DraI-digested fragment of mtDNA with two-dimensional agarose gel electrophoresis. (a) A schematic drawing of the fragment visualised by Southern hybridisation. The non-replicating molecules (1N spot) and replication intermediates (Y arc) are indicated. The molecule structure of the DraI-digested mtDNA replication intermediates at 3 different positions of Y arc is drawn in the insets with a black bar as non-replicating portion and grey bars as replicated portions of the fragment. The apex region of the Y arc used for the quantification is indicated as a grey rectangle and the region of 1N spot used for the quantification as a grey dotted circle. More information on the fragment is provided in Supplementary Fig. 1B. (b–e) The DraI-digested mtDNA fragment of scramble (Sc) dsRNA or DNA ligase III-specific (LIII) dsRNA-treated cells without (− S1) (b and c) and with S1 nuclease treatment (+ S1) (d and e). The samples in panels b–e were run in the same second dimension gel and panels b–e were produced from an X-ray film. Panels c′ and e′ are a longer exposure version of panels c and e. (B) Numerical presentation of the Y arc stability against S1 nuclease. The value of (y/1n _[+ S1]_)/(y/1n _[− S1]_) from Sc dsRNA-treated samples is expressed as 100 and the relative value from LIII dsRNA-treated samples was calculated. Data represent the mean of 3 independent transfection experiments ± SEM.

Next, we investigated the replicating mtDNA molecules using a well-established technique of neutral two-dimensional agarose gel electrophoresis (2D-AGE) [27,28]. This technique has been utilised to study DNA replication in different organisms and was recently used to research the mammalian mtDNA replication mechanism [3–8,29]. R2 DNA samples from Sc dsRNA and LIII dsRNA-treated cells were digested with the restriction enzyme DraI, and half of the sample from each condition was further treated with S1 nuclease (see below). The samples were subjected to 2D-AGE and a DraI-digested fragment spanning nucleotides (nt) 12,271–16,010 of mtDNA (Supplementary Fig. 1B) was detected by Southern hybridisation. Besides the 1N spot that is derived from the non-replicating fragment, the Y arc, the product of replication intermediates of strand-coupled DNA replication (see reference [5] for details) was clearly observed (Fig. 2A). The Y arc was not detectable from cells lacking mtDNA (ρ^0^ cells), confirming that the Y arc is derived from mtDNA (Supplementary Fig. 3). The intensity of the Y arc and 1N spot from LIII dsRNA-treated cells was weaker than that from Sc dsRNA-treated cells (Fig. 2A, panels b and c). This result is consistent with the poor recovery of mtDNA copy number in LIII dsRNA-treated cells (Fig. 1C).

To examine whether the ligation of mitochondrial Okazaki fragments becomes inefficient due to the knockdown of DNA ligase III, we further investigated the replicating mtDNA molecules using S1 nuclease. This nuclease is able to cut the strand opposite to a single strand (ss) nick in duplex DNA as well as cleaving ss DNA [30]. If the Okazaki fragments are joined less efficiently in the DNA ligase III-depleted cells than the control cells, replicating mtDNA molecules in the former condition will contain more ss nicks on the lagging strand, and thus be more susceptible to S1 nuclease, than in control cells. This can be examined by studying the reduction of the Y arc signal upon S1 nuclease treatment. When S1 nuclease cuts a replicating ‘arm’ of the Y-shape molecules which form the Y arc in 2D-AGE (Fig. 2A-a), the cleaved molecules will no longer migrate to the same position as the intact Y-shape molecules in the gels of 2D-AGE, resulting in the decrease of the Y arc signal. Whilst S1 nuclease modified the Y arc from both cell conditions, the extent of diminution of the Y arc appeared slightly greater in LIII dsRNA-treated cells (Fig. 2A). To confirm the visual inspection, we analysed the images as follows: the intensity of the apex region of the Y arc, ‘y’, and that of the 1N spot, ‘1n’ (Fig. 2A-a), was quantified by phosphorimaging densitometry and y was normalised against 1n (y/1n) in each image. The extent of the decrease of the Y arc by S1 nuclease was evaluated by calculating the value of y/1n of the S1 nuclease-treated sample (y/1n _[+ S1]_) divided by y/1n of the non-treated (y/1n _[− S1]_) in each dsRNA transfection. Finally, the value of (y/1n _[+ S1]_)/(y/1n _[− S1]_) from Sc dsRNA-treated sample was expressed as 100 and the relative value of that from LIII dsRNA-treated sample was calculated (Fig. 2B). Even though the relative value from DNA ligase III-depleted cells was reduced roughly by 20%, it did not reach the statistical significance. The fork region of replicating DNA molecules, that is, the position where the two parental strands are separated, should contain a short stretch of ss DNA where the parental strands have become unwound but the complementary nascent strands have not yet been synthesised. *In vivo* single-stranded DNA binding proteins coat this region. This ss DNA region may be easily accessed and cleaved by S1 nuclease in our assay, resulting in the corruption of the Y shaped molecules. The likelihood of this occurrence is equal in both Sc dsRNA and LIII dsRNA-treated cells. We consider that cleavage by S1 nuclease at the replication fork explains the decrease of the Y arc intensity in Sc dsRNA-treated cells (compare Fig. 2A, panels b and d) and also contributes substantially to the reduction of Y arc in LIII dsRNA-treated cells, resulting in the modest trend of the difference in the overall sensitivity of the Y arc replication intermediates to S1 nuclease between them (Fig. 2B). A similar interpretation also applies to the data of Fig. 4B. Considering both the delay of the mtDNA copy number recovery (Fig. 1C) and the data of the 2D-AGE analysis together, we propose that DNA ligase III is responsible for the ligation of Okazaki fragments in human mitochondria.

### 3.3 Knockdown of RNase H1, but neither FEN1 nor DNA2, inhibits the recovery of mtDNA copy number

**Fig. 3:**
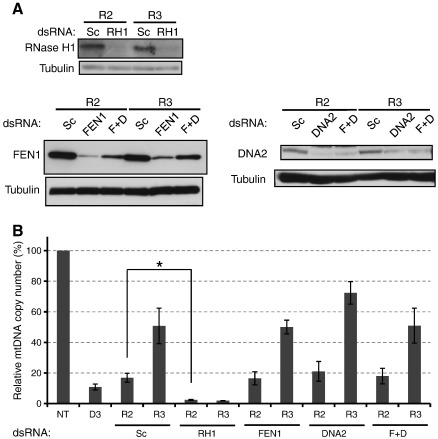
Knockdown of RNase H1, but neither FEN1 nor DNA2 inhibits mtDNA replication. Cells were transiently treated with 2′,3′-dideoxycytidine and transfected with scramble (Sc) dsRNA, RNase H1-specific (RH1) dsRNA, FEN1-specific (FEN1) dsRNA, DNA2-specific (DNA2) dsRNA and both FEN1 dsRNA and DNA2 dsRNA (F + D) as in the diagram of Fig. 1A. (A) Western blot analysis of RNase H1, FEN1 and DNA2 levels. Below each panel Tubulin is shown as loading control. (B) The relative copy number of mtDNA analysed with real-time quantitative qPCR. The interpretation of the graph is as in Fig. 1C. Data represent the mean of 3 independent transfection experiments ± S.E.M. * (*p* < 0.05).

To study whether RNase H1, FEN1 and/or DNA2 are involved in Okazaki fragment processing in mitochondria of human cells, we knocked down these proteins under the conditions described above (Fig. 1A) and examined the changes in mtDNA copy number. Western blot analysis confirmed that RNase H1-specific dsRNA (RH1 dsRNA), FEN1-specific dsRNA (FEN1 dsRNA) and DNA2-specific dsRNA (DNA2 dsRNA) can suppress the expression of the corresponding proteins (Fig. 3A). Analysis of mtDNA copy number with rt-qPCR revealed no significant difference between the recovery profile in cells treated with FEN1 dsRNA or DNA2 dsRNA and those treated with Sc dsRNA (Fig. 3B). In addition, we performed double knockdown of FEN1 and DNA2 as they have been proposed to work synergistically in human mitochondria by *in vitro* assays [16]. The double knockdown did not however delay the recovery of mtDNA copy number. On the contrary, mtDNA copy number in RH1 dsRNA-treated cells on R2 and R3 was even lower than that on D3 (Fig. 3B). This result indicates that depletion of RNase H1 halted mtDNA replication under the present experimental conditions. Additionally, a reduction in mtDNA content in LIII dsRNA- or RH1 dsRNA-treated cells was shown by Southern hybridisation of mtDNA (Supplementary Fig. 4). Note that the protein samples for the DNA2 western blotting were generated in separate transfection experiments as a reliable anti-DNA2 antibody was only obtained at a later stage of the investigation.

### 3.4 Detection of mtDNA replication intermediates and their susceptibility to S1 nuclease under severe mtDNA depletion in RNase H1-depleted cells

**Fig. 4:**
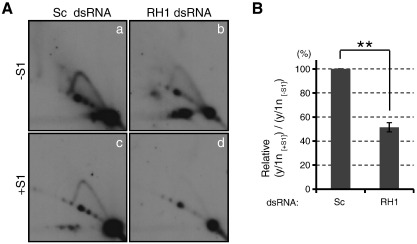
RNase H1 depletion causes enhanced sensitivity to S1 nuclease of mtDNA replication intermediates. (A) Analysis of a *Dra*I-digested mtDNA fragment as in Fig. 2A from cells treated with scramble (Sc) dsRNA or RNase H1-specific (RH1) dsRNA (− S1) (a and b) and those with S1 nuclease treatment (+ S1) (c and d). The images were produced from a single membrane exposed to an X-ray film. (B) Numerical presentation of the Y arc stability against S1 nuclease upon knockdown with RH1 dsRNA. The presentation of the graph is the same as in Fig. 2B. Data represent the mean of 3 independent transfection experiments ± S.E.M. One of the three transfection experiments in Fig. 3B was not used for the production of this graph. Thus the 2D-AGE image from an additional transfection experiment was used instead. ** (*p* < 0.01).

**Fig. 5:**
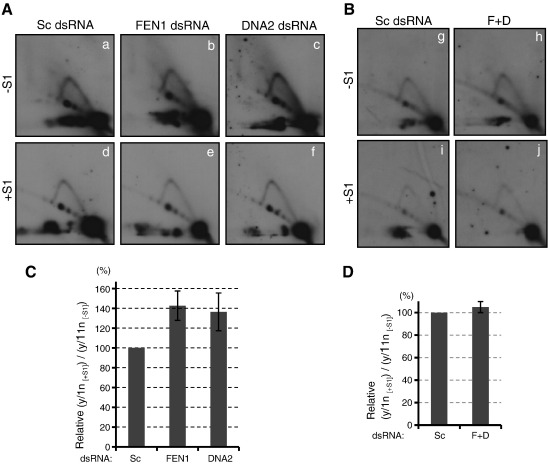
Knockdown of FEN1 and DNA2 does not increase the sensitivity of mtDNA replication intermediates to S1 nuclease. Two-dimensional agarose gel electrophoresis analysis of a DraI-digested mtDNA fragment. Samples were prepared from cells transfected with scramble (Sc) dsRNA, FEN1-specific (FEN1) dsRNA, DNA2-specific (DNA2) dsRNA or both (F + D). (A) Panels a, b and c are samples transfected with Sc, FEN1 and DNA2 dsRNA without S1 nuclease treatment (− S1) respectively, and panels d, e, and f are those with S1 nuclease treatment (+ S1) respectively. Panels a–f were produced from an X-ray film. (B) Panels g and h are samples with the control and double knockdown of FEN1 and DNA2 without S1 nuclease treatment (− S1) respectively, and panels i and j are those with S1 nuclease treatment (+ S1) respectively. Panels g–j were produced from an X-ray film. (C and D) Numerical presentation of the Y arc stability against S1 nuclease upon knockdown with FEN1 dsRNA, DNA2 dsRNA (C) and the double knockdown of FEN1 and DNA2 (F + D) (D). The presentation of the graph is the same as in Fig. 2B. Data represent the mean of 3 independent transfection experiments ± S.E.M. (C) and the mean of 2 independent transfection experiments with the bars representing the range of the values (D).

To investigate the effect of RNase H1 knockdown further, 2D-AGE analysis was performed with DNA samples harvested on R2. Despite the dramatic decrease of mtDNA copy number (Fig. 3B), the Y arc was relatively easily detected in RH1 dsRNA-treated cells (Fig. 4A, panel b). This implies that the mitochondrial replisomes are stalled on replicating molecules upon RNase H1 depletion, resulting in a ‘freeze’ of the replication intermediates. Intriguingly, the Y arc from the RH1 dsRNA-treated sample was more susceptible to S1 nuclease compared to that from the Sc dsRNA-treated sample, suggesting an increase of ss nicks and/or gaps on the replication intermediates from the strand-coupled DNA synthesis mode (Fig. 4). These data strongly suggest that RNase H1 is an indispensable protein for Okazaki fragment processing in human mtDNA replication. In contrast, no apparent increase of S1 nuclease susceptibility of the Y arc from the R2 samples was observed upon knockdown of FEN1 or DNA2, or simultaneous knockdown of both (Fig. 5).

## 4 Discussion

In this study, we suppressed the expression of target proteins by dsRNA transfection and investigated the effect on mtDNA replication after transient depletion of mtDNA, when mtDNA is amplified mainly through the strand-coupled DNA synthesis mode to restore the mtDNA copy number [3,5]. Under these conditions, the involvement of the proteins in RNA primer removal and DNA ligation was examined. Knockdown of a protein with dsRNA transfection can be conducted much less laboriously than creation of a system lacking expression of the protein, such as a knockout mouse. Also, since we can investigate the effect of knockdown of the target proteins on mtDNA replication within a short duration in the dsRNA-induced knockdown system, any indirect effect on mtDNA as a result of prolonged knockout/knockdown of the protein of interest can be avoided. Therefore, although studies on the target protein in the context of a life process such as development are not possible in our assay system, this system is suitable for investigating the function of the protein in the process of mtDNA replication at the molecular level.

Campbell and colleagues observed a decrease of mtDNA copy number and an increase of lesions such as ss nicks in mtDNA in cell clones that constitutively expressed antisense RNA against DNA ligase III mRNA [22] or catalytically inactive DNA ligase III mutants [23]. These observations could be the consequence of delayed ligation in mtDNA replication, yet the authors' experiments did not appear to address this point directly. The long term constitutive expression of either antisense RNA or mutant proteins in the cell clones made it difficult to examine DNA ligase III in the processes of DNA repair and DNA replication separately. On the other hand, in our study the acute knockdown of DNA ligase III during mtDNA amplification following transient mtDNA depletion enabled us to focus on the function of the protein in the mtDNA replication process. Here we demonstrated that the presence of DNA ligase III is crucial for the restoration of mtDNA copy number after mtDNA depletion. Further, depletion of DNA ligase III seemed to delay the elimination of nicks on the replicating mtDNA molecules. These findings support the role of DNA ligase III as the replicative DNA ligase in human mtDNA replication and suggest that DNA ligase III functions in the maturation process of Okazaki fragments. Our conclusion is consistent with both the current understanding that DNA ligase III is the only DNA ligase present in human mitochondria [21,22], and the recent publications which indicated that DNA ligase III is the replicative DNA ligase in mouse mitochondria [31,32].

We found that the copy number of mtDNA in R2 and R3 samples of RH1 dsRNA-treated cells became significantly lower than that in D3 samples, yet the mtDNA replication intermediates derived from the strand-coupled DNA synthesis were relatively easily detected in the R2 sample without S1 nuclease treatment. These results together suggest that DNA synthesis in the elongation process of the strand-coupled DNA replication was halted completely upon RNase H1 depletion. Furthermore, S1 nuclease treatment implied an increase of ss nicks and/or gaps on the mtDNA replication intermediates from RNase H1-depleted cells. Considering these findings, we propose the following scenario: RNase H1 depletion impaired the RNA primer removal step, leaving unprocessed RNA primer present which subsequently prevented the ligation of Okazaki fragments, and resulted in nicks/or gaps between them. Collectively, this defective process, caused by the lack of RNase H1, ceased the progress of DNA synthesis in the strand-coupled DNA replication and prevented restoration of mtDNA copy number after transient mtDNA depletion. The extent of inhibition of mtDNA recovery in RNase H1-depleted cells was more severe than in DNA Ligase III-depleted cells. This may be due to a difference in the efficiency of knockdown of RNase H1 and DNA ligase III or in their abundance in mitochondria. We thus propose that RNase H1 is indispensable for the maturation process of Okazaki fragments in human mtDNA replication (see Supplementary Fig. 5 for a possible model of the maturation process).

It was proposed that in the nuclear DNA2/FEN1 pathway of Okazaki fragment processing a long flap containing RNA primer and the 5′ end DNA portion of the Okazaki fragment is generated by a strand displacement activity of DNA polymerase (pol) δ which is synthesising the next Okazaki fragment. The flap is then cleaved by DNA2 nuclease activity, leaving a short flap behind, which will be trimmed by FEN1 and the 5′ end may be ligated with the 3′ end of the next Okazaki fragment [10,12]. In the nucleus, since the 5′ end DNA portion of Okazaki fragments is generated by pol α which lacks a proofreading function (and the rest of Okazaki fragments by DNA pol δ), the DNA2/FEN1 pathway would be important for the removal of the 5′ end DNA portion to maintain genome stability [12]. This removal of the 5′ end DNA portion is likely to be unnecessary in mammalian mitochondria since pol γ, the only replicative DNA polymerase in the mitochondria, has a 3′–5′ exonuclease activity [33] and may therefore synthesise the entire Okazaki fragment during mtDNA replication. The DNA2/FEN1 pathway might therefore be functionally dispensable and may be a minor pathway in the human mitochondrial replication system. That knockdown of DNA2 or both DNA2 and FEN1 in the present investigation did not induce even a modest delay in the recovery of mtDNA copy number after transient mtDNA depletion or any increase of susceptibility of the replication intermediates to S1 nuclease fits well with the above hypothesis. Also, the dramatic effects of the RNase H1 depletion suggest that most of the RNA primers are processed by this nuclease *in vivo* and that DNA2 does not complement the role of RNase H1. We thus infer that DNA2 is specialised in DNA repair in human mitochondria. However, it is possible that the extent of the reduction in the level of expression of DNA2 in this study was insufficient to affect mtDNA replication, or that our analysis methods were not sensitive enough to detect defects in mtDNA replication upon DNA2 knockdown.

It is considered that in the RNase H/FEN1 pathway of nuclear Okazaki fragment processing RNase H2 cleaves the RNA primer but leaves a single ribonucleotide at 5′ end of DNA, and that this ribonucleotide is removed by FEN1 [10]. A previous study suggested that RNase H1 leaves two ribonucleotides at least at 5′ end of DNA [34]. Thus one or more additional enzymes, such as FEN1, should remove the remaining ribonucleotides in mitochondria as well. A lack of inhibition of mtDNA replication upon FEN1 depletion observed in this study was thus surprising, as residual ribonucleotides could disturb the Okazaki fragment ligation *in vivo*. Our result could be explained by the presence of a backup nuclease. A potential candidate, exonuclease 5, was reported to exist in yeast mitochondria and a putative human homolog has been found as an uncharacterised open reading frame [35]. On the other hand, exonuclease 1 is suggested to be a backup protein of FEN1 in nuclear DNA replication system [10,12]. Another possible explanation of our observation in FEN1 knockdown would be that the reduction in FEN1 expression levels was not sufficient to affect mtDNA replication.

In the nucleus, DNA ligase I joins Okazaki fragments [13] and RNase H2 participates in Okazaki fragment processing in the RNase H/FEN1 model [10]. Our findings thus suggest that DNA ligase III and RNase H1 substitute for DNA ligase I and RNase H2 respectively in the human mitochondrial replication system.

The following are the supplementary materials related to this article.

Supplementary Fig. 1 Mitochondrial DNA (mtDNA) replication after transient 2′,3′-dideoxycytidine (ddC) treatment.

Supplementary Figs 2 Western blot analysis of DNA ligase III levels in crude mitochondria.

Supplementary Fig. 3 Absence of the Y arc from cells lacking mtDNA.

Supplementary Fig. 4 Southern hybridisation analysis of mtDNA content.

Supplementary Fig. 5 A possible model for the lagging strand synthesis in the strand-coupled DNA synthesis mode in human mitochondria.

Supplementary materials.
