# Supplementary material for: Involvement of DNA ligase III and ribonuclease H1 in mitochondrial DNA replication in cultured human cells
Source: Biochim Biophys Acta. 2011 Dec;1813(12):2000–7. doi: 10.1016/j.bbamcr.2011.08.008 (PMC3223524; doi:10.1016/j.bbamcr.2011.08.008)
Supplement: Supplementary Fig. 1 — Mitochondrial DNA (mtDNA) replication after transient 2′,3′-dideoxycytidine (ddC) treatment. [file mmc1.ppt]

## Slide 1
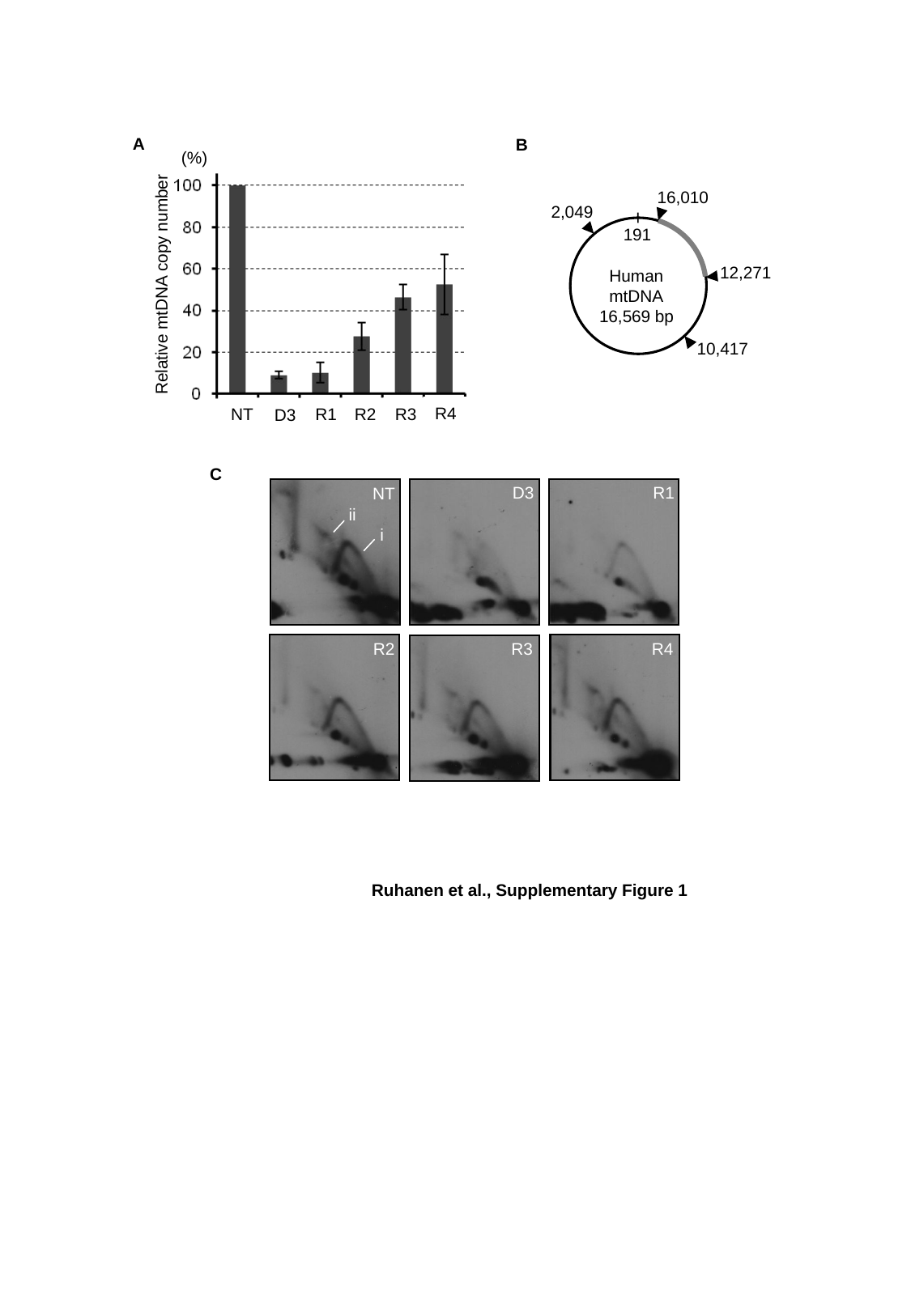

A
B
(%)
16,010
2,049
191
12,271
Human
mtDNA
16,569 bp
Relative mtDNA copy number
10,417
R4
NT
R1
R2
R3
D3
C
D3
R1
NT
ii
i
R2
R3
R4
Ruhanen et al., Supplementary Figure 1
