# Supplementary material for: Involvement of DNA ligase III and ribonuclease H1 in mitochondrial DNA replication in cultured human cells
Source: Biochim Biophys Acta. 2011 Dec;1813(12):2000–7. doi: 10.1016/j.bbamcr.2011.08.008 (PMC3223524; doi:10.1016/j.bbamcr.2011.08.008)
Supplement: Supplementary Figs 2 — Western blot analysis of DNA ligase III levels in crude mitochondria. [file mmc2.ppt]

## Slide 1
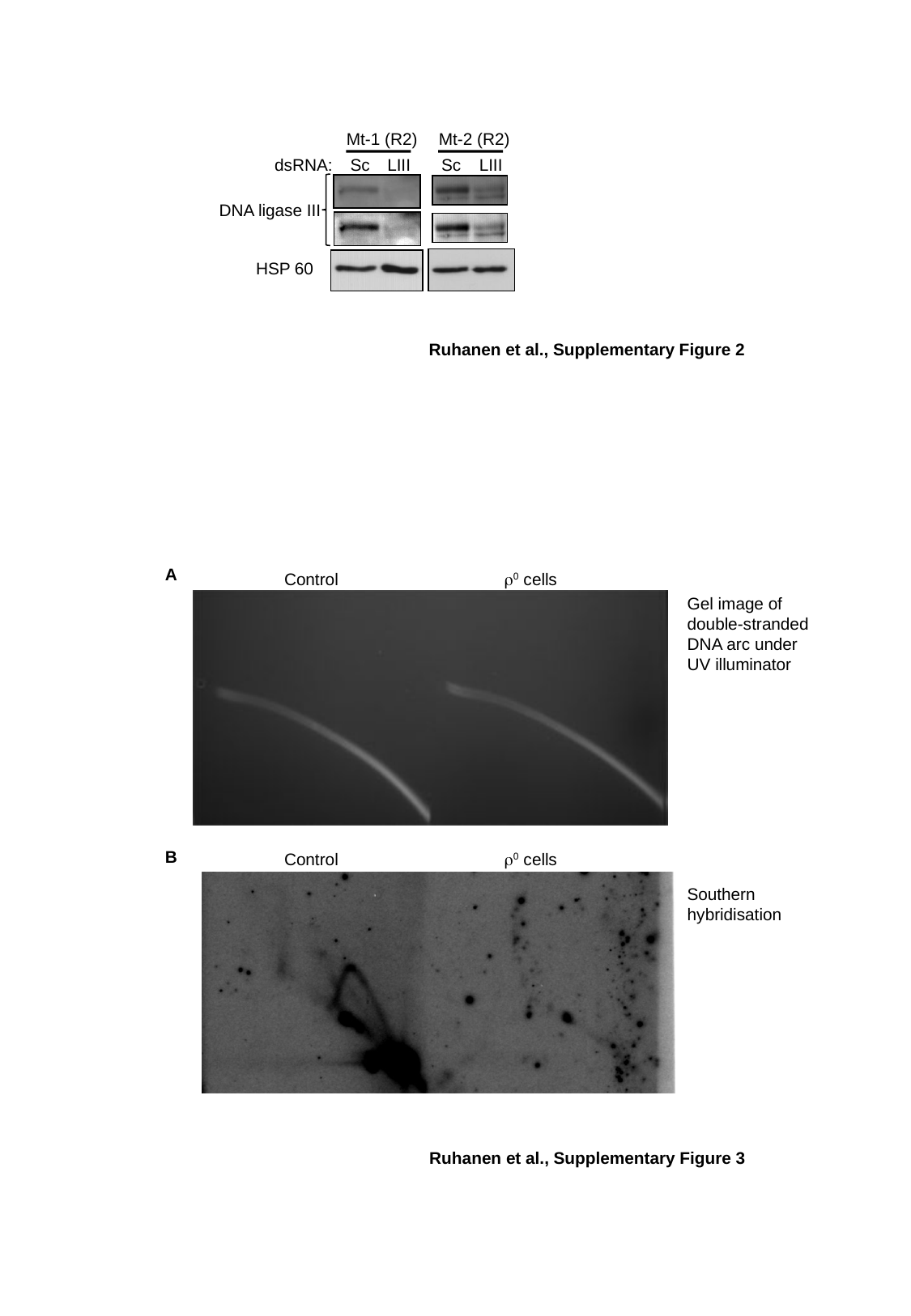

Mt-1 (R2)
Mt-2 (R2)
dsRNA:
Sc
Sc
LIII
LIII
DNA ligase III
HSP 60
Ruhanen et al., Supplementary Figure 2
A
Control
0 cells
Gel image of
double-stranded DNA arc under UV illuminator
B
Control
0 cells
Southern hybridisation
Ruhanen et al., Supplementary Figure 3
