# Supplementary figures and images for: Involvement of DNA ligase III and ribonuclease H1 in mitochondrial DNA replication in cultured human cells
Source: Biochim Biophys Acta. 2011 Dec;1813(12):2000–7. doi: 10.1016/j.bbamcr.2011.08.008 (PMC3223524; doi:10.1016/j.bbamcr.2011.08.008)

## Slide 1
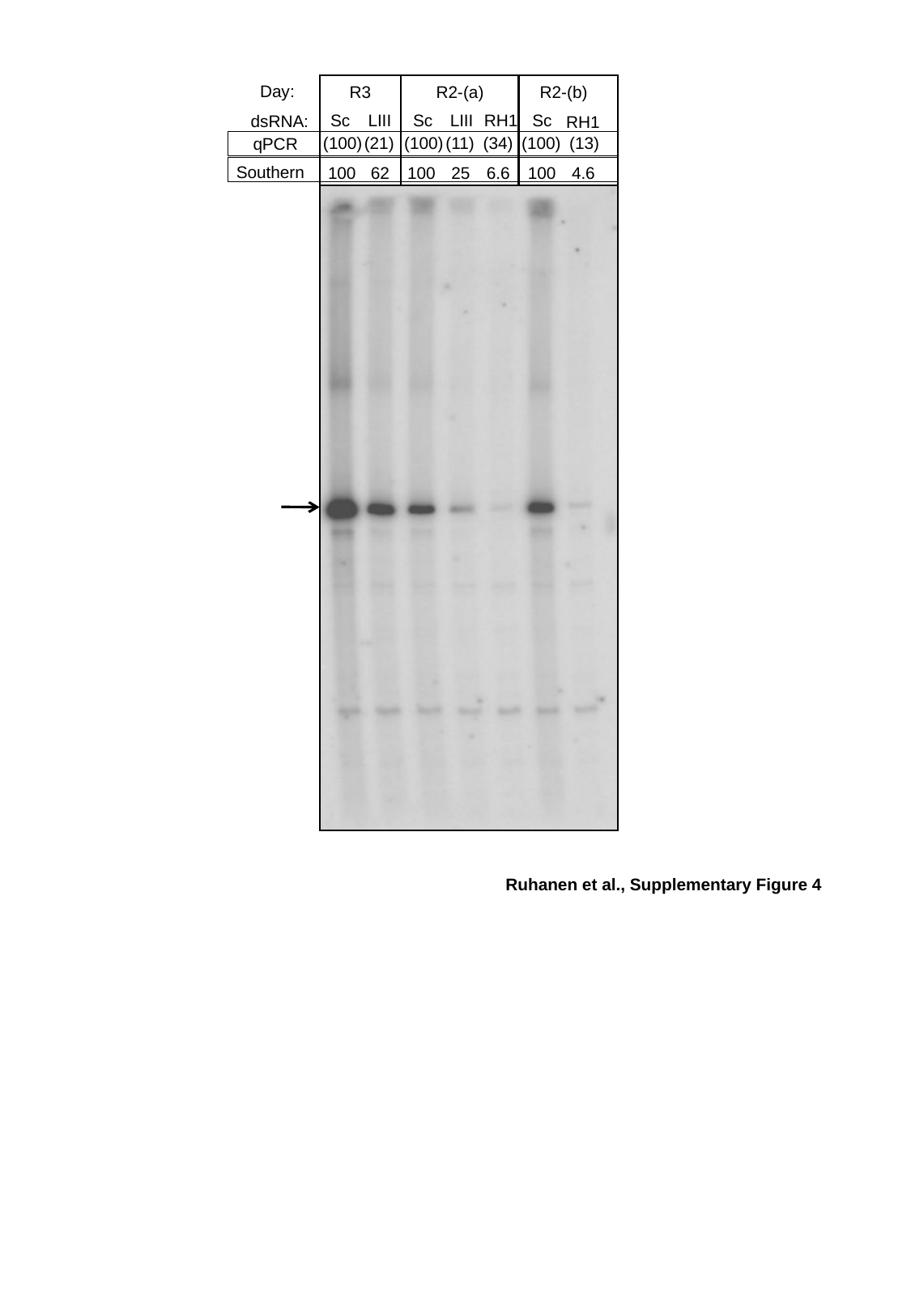

Day:
R2-(a)
R2-(b)
R3
Sc
LIII
Sc
LIII
RH1
Sc
dsRNA:
RH1
(21)
(100)
(100)
(11)
(34)
(100)
(13)
qPCR
Southern
62
100
100
25
6.6
100
4.6
Ruhanen et al., Supplementary Figure 4

Supplement: Supplementary Fig. 4 — Southern hybridisation analysis of mtDNA content. [file mmc3.ppt]
