# Supplementary material for: Involvement of DNA ligase III and ribonuclease H1 in mitochondrial DNA replication in cultured human cells
Source: Biochim Biophys Acta. 2011 Dec;1813(12):2000–7. doi: 10.1016/j.bbamcr.2011.08.008 (PMC3223524; doi:10.1016/j.bbamcr.2011.08.008)
Supplement: Supplementary Fig. 5 — A possible model for the lagging strand synthesis in the strand-coupled DNA synthesis mode in human mitochondria. [file mmc4.ppt]

## Slide 1
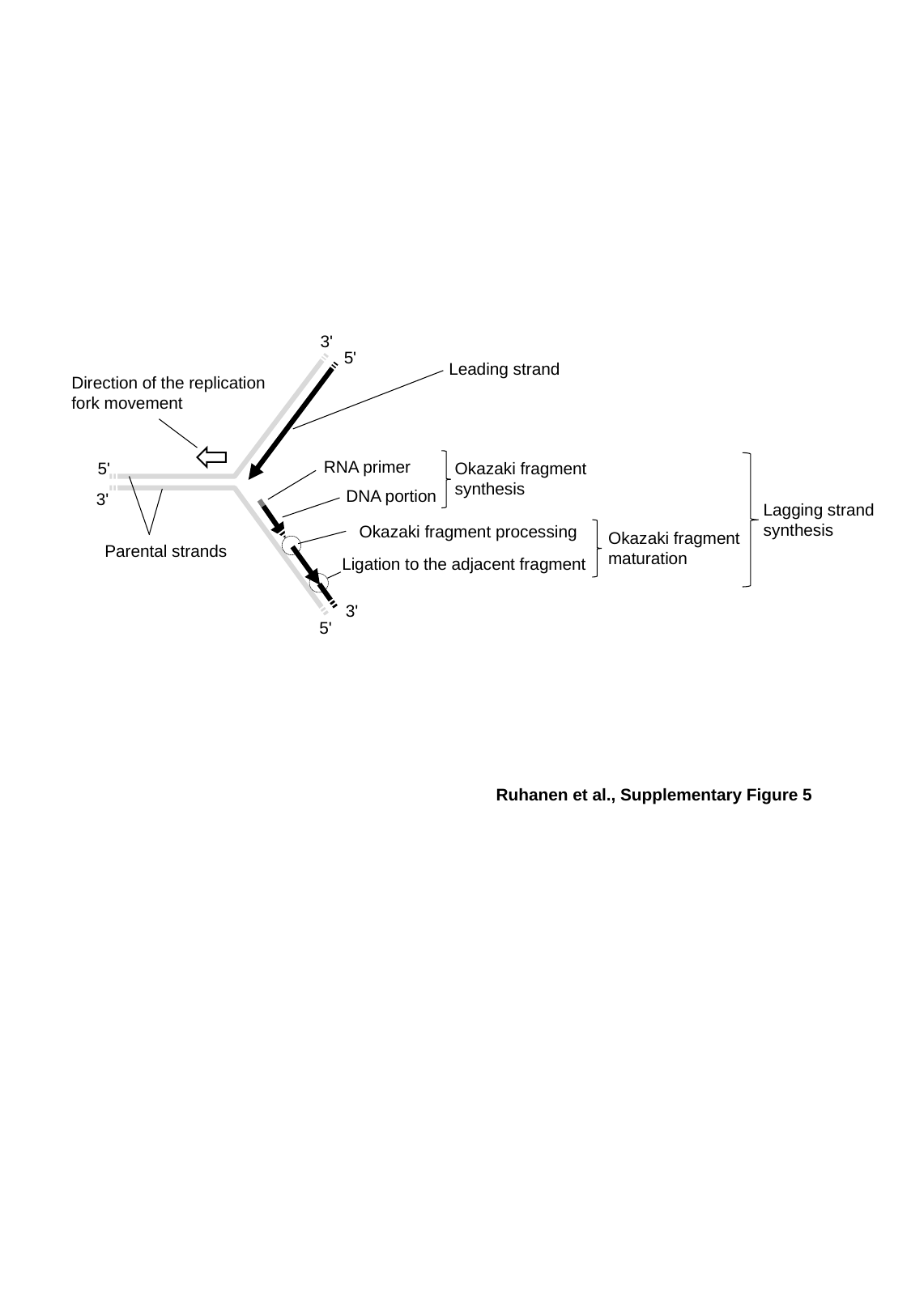

3'
5'
Leading strand
Direction of the replication
fork movement
RNA primer
Okazaki fragment
synthesis
5'
DNA portion
3'
Lagging strand
synthesis
Okazaki fragment processing
Okazaki fragment
maturation
Parental strands
Ligation to the adjacent fragment
3'
5'
Ruhanen et al., Supplementary Figure 5
