# Supplementary material for: Involvement of DNA ligase III and ribonuclease H1 in mitochondrial DNA replication in cultured human cells
Source: Biochim Biophys Acta. 2011 Dec;1813(12):2000–7. doi: 10.1016/j.bbamcr.2011.08.008 (PMC3223524; doi:10.1016/j.bbamcr.2011.08.008)
Supplement: Supplementary file 5 — Supplementary materials. [file mmc5.doc]

**Involvement of DNA ligase III and Ribonuclease H1 in Mitochondrial DNA Replication in Cultured Human Cells**

**Heini Ruhanen, Kathy Ushakov and Takehiro Yasukawa**

*The Wolfson Institute for Biomedical Research, University College London, Gower Street, London, WC1E 6BT, UK*

**SUPPLEMENTARY INFORMATION**

**Figure Legends**

**Supplementary Figure 1. Mitochondrial DNA (mtDNA) replication after transient 2’,3’-dideoxycytidine (ddC) treatment.**

(**A**) Recovery profile of mtDNA copy number. Cells were incubated in 25 μM ddC-containing medium for 3 days and then washed with normal medium, followed by further culture for 4 days in normal medium, without dsRNA transfection. The amount of mtDNA was analysed with real-time quantitative PCR. NT, cells cultured for 3 days without ddC; D3, cells cultured for 3 days with ddC; R1-R4, cells cultured in normal medium for 1 to 4 days after 3 day incubation with ddC. The graph is the mean of two independent experiments and the bars represent the range of the values. (**B**) Schematic map of human mtDNA. DraI restriction sites are indicated by arrowheads with the nucleotide (nt) number of the sites. The position of nt 191 is also shown. The probe used in this study hybridised to a region in the DraI fragment of nt 12,271-16,010 (highlighted as a thick gray line) in Figures 2, 4 and 5 in the main manuscript and Supplementary Figure 1. (**C**) Two-dimensional agarose gel electrophoresis analysis of the mtDNA replication intermediates. In NT sample, two arcs were readily detected. The right arc (i), called the Y arc stemming from the 1N spot is derived from strand-coupled DNA synthesis and the left arc (ii) from RITOLS replication (see Figure 1 in Reference ). The Y arc is the major replication intermediates in the samples R1-3, suggesting that mtDNA is replicated mainly via the strand-coupled DNA synthesis mode of replication at the time of sample collection in the main text.

**Supplementary Figure 2. Western blot analysis of DNA ligase III levels in crude mitochondria.**

Western blot analysis of DNA ligase III levels in crude mitochondria prepared from cells treated with Sc dsRNA or LIII dsRNA and harvested on R2. Crude mitochondrial fraction was prepared using Mitochondria Isolation Kit for Cultured Cells (Thermo Scientific). The top panel shows the DNA ligase III band and the middle panel is a digitally enhanced image of the top panel. Heat shock protein 60 (HSP 60) was detected with an anti-HSP 60 antibody (sc-13115, Santa Cruz Biotechnology) and used as a loading control (bottom panel). Two independent transfection experiments were performed (Mt-1 and Mt-2). More materials were loaded in the set of Mt-2.

**Supplementary Figure 3. Absence of the Y arc from cells lacking mtDNA**

To confirm that the Y arc detected in 2D-AGE analysis in this study is derived from mtDNA, the 2D-AGE experiment was performed using HeLa-derived ρ^0^ cell line EB8 that is lacking mtDNA (a kind gift from Shigeo Ohta, Nippon Medical School, Japan). Total DNA prepared from control and EB8 cells was digested with DraI and subjected to 2D-AGE and Southern hybridisation. (**A)** UV transilluminator image of 2D-AGE gel after the electrophoresis of the second dimension gel. The double-stranded DNA arcs (the series of linear DNA molecules) were visualised as the second dimension gel contains ethidium bromide. Comparable intensity of the arcs from the control and ρ^0^ samples indicated the similar loading of DNA between them. (**B)** Southern hybridisation image of the DraI-digested mtDNA from the control cells that from ρ^0^ cells. The DNA on the gel in (**A)** was blotted onto a solid support and Southern hybridisation with the probe covering nt 12,981-13,384 of mtDNA was performed as described in 2.4. in the main text. The Y arc was not detected from the ρ^0^ sample, confirming that the Y arc is derived from mtDNA replicating molecules.

**Supplementary Figure 4. Southern hybridisation analysis of mtDNA content.**

Approximately 1.5 μg of the samples were digested with PvuII and electrophoresed in agarose gel. The samples were total DNA preparation without RNase treatment and thus contain RNA as well. The gel was blotted onto a solid support, subjected to Southern hybridisation with the probe covering nt 12,981-13,384 of mtDNA and exposed to a phosphorimaging plate. The intensity of the bands corresponding to the linearlised mtDNA (indicated by an arrow) was quantified (human mtDNA contains a single PvuII restriction site). A set of R3 samples and two different sets of R2 samples (R2-(a) and R2-(b)) were analysed. In each set of the transfection experiment the value of mtDNA sample in Sc dsRNA-treated sample was set as 100 and the relative values in either LIII dsRNA or RH1 dsRNA-treated samples were calculated and showed above the gel image with an indication of ‘Southern’. The values in the parathness with ‘qPCR’ are calculated from the qPCR result of the samples.

**Supplementary Figure 5. A possible model for the lagging strand synthesis in the strand-coupled DNA synthesis mode in human mitochondria**

By analogy with the nuclear DNA replication mechanism, the lagging strand synthesis in the strand-coupled DNA synthesis mode in human mitochondria must be achieved by the synthesis of Okazaki fragments and subsequent maturation. The maturation process, in which the Okazaki fragments are joined together to form a continuous nascent strand, should require two sequential steps. The first step is removal of RNA primer, called Okazaki fragment processing. The second step is ligation of the processed 5' end of the Okazaki fragment and the 3' end of the next Okazaki fragment. We propose that RNase H1 and DNA ligase III are involved in the Okazaki fragment processing and ligation, respectively, in human mitochondria. DNA synthesis in both the leading and lagging strands must be performed solely by DNA polymerase γ, the only known replicative DNA polymerase in human mitochondria.

It is not known how the RNA primer is provided in the lagging strand synthesis in the mitochondrial strand-coupled DNA synthesis mode. One obvious possibility is that the mitochondrial system employs a mechanism similar to that of other replication systems, in which a short RNA molecule is synthesised for every Okazaki fragment to prime DNA synthesis. In support of this idea, mitochondrial primase activities have previously been reported . Also, it was recently shown that mitochondrial RNA polymerase is capable of generating short stretches of RNA . It is also possible that nascent light (L)-strand RNA in the replication intermediates of the RITOLS mode could somehow provide multiple short RNA molecules suitable for the priming of DNA synthesis, as has been speculated previously .

**Additional information on the 2D-AGE images**

Within each set of panels in Figure 2A (b, c, d and e), Figure 4A (a, b, c and d), Figure 5A (a, b, c, d, e and f) and Figure 5B (g, h, i and j), the intensity of the images between the panels can be compared, as they were produced from a single membrane. On the other hand, a direct comparison of the images (intensities) of the panels between different Figures is not suitable, as they were from different membranes.

**References**

[1] T. Yasukawa, M.Y. Yang, H.T. Jacobs, I.J. Holt, A bidirectional origin of replication maps to the major noncoding region of human mitochondrial DNA, Mol. Cell, 18 (2005) 651-662.

[2] J. Hayashi, S. Ohta, A. Kikuchi, M. Takemitsu, Y. Goto, I. Nonaka, Introduction of disease-related mitochondrial DNA deletions into HeLa cells lacking mitochondrial DNA results in mitochondrial dysfunction, Proc. Natl. Acad. Sci. USA., 88 (1991) 10614-10618.

[3] B.J. Ledwith, S. Manam, G.C. Van Tuyle, Characterization of a DNA primase from rat liver mitochondria, J. Biol. Chem., 261 (1986) 6571-6577.

[4] T.W. Wong, D.A. Clayton, DNA primase of human mitochondria is associated with structural RNA that is essential for enzymatic activity, Cell, 45 (1986) 817-825.

[5] S. Wanrooij, J.M. Fuste, G. Farge, Y. Shi, C.M. Gustafsson, M. Falkenberg, Human mitochondrial RNA polymerase primes lagging-strand DNA synthesis in vitro, Proc. Natl. Acad. Sci. USA., 105 (2008) 11122-11127.

[6] T. Yasukawa, A. Reyes, T.J. Cluett, M.Y. Yang, M. Bowmaker, H.T. Jacobs, I.J. Holt, Replication of vertebrate mitochondrial DNA entails transient ribonucleotide incorporation throughout the lagging strand, EMBO J., 25 (2006) 5358-5371.
